# Longitudinal Changes in Food Addiction Symptoms and Body Weight among Adults in a Behavioral Weight-Loss Program

**DOI:** 10.3390/nu12123687

**Published:** 2020-11-29

**Authors:** Eliza L. Gordon, Lisa J. Merlo, Patricia E. Durning, Michael G. Perri

**Affiliations:** 1 Department of Clinical and Health Psychology, University of Florida, P.O. Box 100165, Gainesville, FL 32610, USA; pdurning@phhp.ufl.edu (P.E.D.); mperri@phhp.ufl.edu (M.G.P.); 2 Division of Physical Medicine & Rehabilitation, University of Utah, 30 N 1900 E (Rm 1B620), Salt Lake City, UT 84132, USA; 3 Department of Psychiatry, McKnight Brain Institute, University of Florida College of Medicine, L4-100K, P.O. Box 100256, Gainesville, FL 32611, USA; lmerlo@ufl.edu

**Keywords:** obesity, food addiction, weight loss, treatment, food

## Abstract

Interest in food addiction (FA) has increased, but little is known about its clinical implications or potential treatments. Using secondary analyses from a randomized controlled trial, we evaluated the associations between changes in FA, body weight, and “problem food” consumption during a 22-month behavioral weight-loss program consisting of an initial four-month in-person intervention, 12-month extended-care, and six-month follow-up (*n* = 182). Food addiction was measured using the Yale Food Addiction Scale. “Problem foods” were identified from the literature and self-reporting. Multilevel modeling was used as the primary method of analysis. We hypothesized that reductions in problem food consumption during the initial treatment phase would be associated with long-term (22-month) FA reductions. As expected, we found that reductions in problem foods were associated with greater initial reductions in FA symptoms; however, they were also associated with a sharper rebound in symptoms over time (*p* = 0.016), resulting in no significant difference at Month 22 (*p* = 0.856). Next, we hypothesized that long-term changes in FA would be associated with long-term changes in body weight. Although both FA and weight decreased over time (*p*s < 0.05), month-to-month changes in FA were not associated with month-to-month changes in weight (*p* = 0.706). Instead, higher overall FA (i.e., mean scores over the course of the study) were associated with less weight loss (*p* = 0.008) over time. Finally, we hypothesized that initial reductions in problem food consumption would be associated with long-term reductions in weight, but this relationship was not significant (*p*s > 0.05). Given the complexity of the findings, more research is needed to identify interventions for long-term changes in FA and to elucidate the associations between problem foods, FA, and weight.

## 1. Introduction

Prior research has suggested that certain foods (e.g., processed foods high in fat and/or sugar) and eating behaviors (e.g., binge eating) can be associated with addiction-like symptoms. Neurological, genetic, and psychological similarities have been observed between problematic eating behaviors and symptoms of substance use disorders (e.g., excessive consumption, cravings, preoccupation, unsuccessful attempts to limit use of the substance) [1,2,3,4,5,6,7,8,9,10]. High-fat and/or high-sugar processed foods, such as ice cream, pizza, potato chips, or chocolate, are most commonly associated with addiction-like changes both behaviorally and neurobiologically [9,11,12]. For example, Gearhardt et al. [12] found that individuals who reported experiencing DSM-IV substance dependence symptoms toward food had greater activation in the anterior cingulate cortex, medial orbitofrontal cortex, and amygdala (brain areas implicated in substance dependence) in response to a chocolate milkshake.

In 2009, Gearhardt et al. [13] published the Yale Food Addiction Scale (YFAS), a validated self-report questionnaire that adapts the DSM-IV substance dependence criteria toward “certain foods” for which respondents may have difficulty controlling their intake. Using this scale, Schulte and Gearhardt [14] estimated that approximately 15% of adults in the United States met the YFAS criteria for food addiction (FA; ≥3 symptoms, plus distress/impairment), with higher prevalence among adults with obesity (19%). In addition to obesity, FA symptoms have been associated with increased risk for disordered eating [8,15,16,17], depression [15,16,17,18], emotional eating [15,17], impulsivity [16], lower self-esteem [16], and poorer quality of life [19].

Despite increased empirical interest in FA, research toward evidence-based treatment is inchoate. A systematic review by Cassin et al. [20] found only eight studies related to FA treatment and concluded that there is not sufficient evidence to support any specific intervention. There is a clear need for research toward evidence-based interventions if FA continues to present as a unique problem [21,22,23]. Potential treatments could draw on successful approaches used in the substance use disorder and obesity treatment literature. For example, Vella and Pai [24] proposed that techniques commonly used in substance use disorder and obesity treatment—such as problem solving, stimulus control, and cognitive behavioral approaches—could help treat FA by reducing impulsivity, building positive coping skills, and improving distress tolerance. 

Behavioral weight-loss treatments represent a logical next step toward identifying evidence-based treatments for FA due to their strong theoretical base, effectiveness in treating obesity, and similarity to substance use disorder treatment. Grounded in cognitive-behavioral theory, these interventions aim to produce healthy weight loss primarily by decreasing caloric consumption, improving diet quality, and increasing physical activity [25]. Often provided in a group setting, these interventions can be a source of health-behavior-specific social support, which is vital to long-term recovery from drug addiction [26], and possibly FA [27]. 

To our knowledge, only two studies have evaluated changes in FA symptoms among adults participating in behavioral weight-loss programs. In a sample of 90 women, Sawamoto et al. [28] found that “successful” participants (i.e., those who maintained a 10% weight loss at 12- and 24-month follow-ups) in a seven-month behavioral weight-loss program reported fewer symptoms of FA post-treatment compared to “unsuccessful” participants, despite no differences in symptoms at baseline. However, the authors did not report whether changes in FA were statistically significant. Chao et al. [29] analyzed changes in weight and FA symptoms among a sample of 178 adults participating in a 14-week behavioral weight-loss program. They found that, although overall YFAS scores significantly decreased from pre- to post-intervention, neither changes in YFAS scores nor baseline YFAS “diagnosis” significantly predicted weight loss. Taken together, findings from these studies appear to suggest that FA symptoms may decrease in behavioral weight-loss programs.

The current study aimed to describe the associations between early changes in “problem food” consumption, long-term changes in FA symptoms, and long-term weight change. Analyses were conducted using data collected from a multi-site behavioral weight-loss randomized control trial that consisted of a four-month in-person treatment phase (Phase 1; Months 0–4), followed by a 12-month extended-care phase (Phase 2; Months 4–16) and a six-month follow-up phase (Phase 3; Months 16–22). Specifically, we aimed (1) to identify associations between initial (Phase 1) changes in “problem food” consumption (e.g., high-sugar/high-fat processed foods recorded in participant dietary logs) and long-term changes in FA symptoms, and (2) to identify the associations between initial changes in “problem food” consumption, long-term FA symptoms, and long-term weight loss. We hypothesized that (a) Phase 1 reductions in “problem foods” would be associated with a long-term reduction in FA symptoms (Months 0–22), (b) long-term changes in FA symptoms would be associated with long-term changes in body weight, and (c) Phase 1 reductions in problem food consumption would be associated with long-term reductions in body weight.

## 2. Materials and Methods

The current paper describes a secondary data analysis of data from the Rural Lifestyle Eating and Activity Program (Rural LEAP) [30]. The Rural LEAP project was a randomized controlled trial comparing the effects of three strategies for long-term weight management among 528 women and men (ages 21–75; body mass indexes (BMIs) between 30–45) living in rural north Florida. Approval was obtained from the University of Florida Institutional Review Board (IRB), and all participants gave written informed consent. Participants attended a weekly, in-person behavioral weight-loss program for the first four months of treatment (Phase 1). Those who completed Phase 1 with ≥50% attendance were randomly assigned to 12 months of extended care (Phase 2) delivered via individual or group telephone counseling or an education control program delivered via email. All participants received 18 modules with recommendations for maintaining lost weight. In the phone-based conditions, health coaches provided participants with 18 individual or group sessions focused on problem solving of obstacles to the maintenance of weight loss. Phase 3 (Months 16–22) was a no-contact follow-up period for participants to practice the strategies on their own. Assessments were conducted at baseline (Month 0), post-treatment (Month 4), halfway through the extended-care phase (Month 10), post-extended care (Month 16), and after the final no-contact phase (Month 22). 

### 2.1. Participants

**Figure 1:**
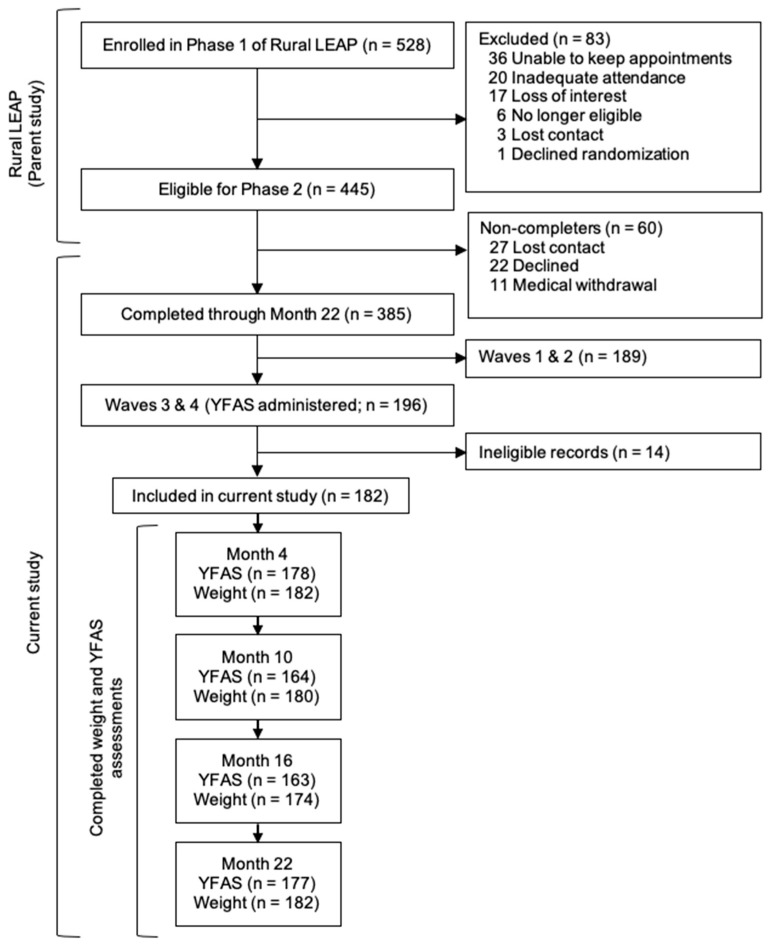
Flow diagram of participants.

The sample included adult men and women with BMIs between 30–45 kg/m^2^ and without medical contraindications for weight loss (see prior publications [30,31] for specific inclusion and exclusion criteria). Although four cohorts (“waves”) of volunteers participated in the Rural LEAP program, the YFAS was not added to the study until the third and fourth cohorts; thus, the current study only included participants who completed the program during Waves 3 and 4 (*n* = 196). In addition, the current study excluded participants if they failed to complete eligible dietary records at the end of Month 4 (i.e., ≥3 days recorded per week; complete information on food types/amounts) or if they did not complete the 22-month assessment. This resulted in a sample size of 182 participants for the current study (see Figure 1 for a flow diagram).

### 2.2. Main Variables

#### 2.2.1. Problem foods 

“Problem foods” (i.e., foods more likely to be associated with addiction-like symptoms) were self-identified at baseline using participants’ responses to the YFAS. Combined with information from prior literature (e.g., [9,11,32,33,34]), these responses were used to create a combined “problem foods” variable, which included the following food categories from the USDA’s “What We Eat In America” survey [35]: pizza, burgers, savory snacks, sweet bakery products, chocolate, candy, ice cream and frozen desserts, fried potatoes, diet soda, sweetened beverages, sugars, and sugar substitutes. A “problem food consumption” score was calculated for each participant based on the average number of times per day he or she consumed a food item from this list. This was calculated by summing the total number of times participants consumed any item from the “problem food” list in a week (e.g., first week of Month 0; last week of Month 16) and dividing this sum by the number of days recorded that week (e.g., average daily frequency of problem food consumption = total number of times “problem food” consumed that week/number of days recorded that week). Data on food consumption was extracted from participants’ dietary records, which they were instructed to keep daily during the course of the intervention. Due to low food record completion rates during Phase 2 (approximately 50%), only food logs from Phase 1 were used for the current study. Data extracted from the logs were entered into the secure Research Electronic Data Capture (REDCap) tool [36], and entries were randomly checked for accuracy and consistency. Food records were excluded if they failed to include accurate or complete information (e.g., no quantities or caloric values), if there were less than three complete days recorded per week, or if the participant had abnormal circumstances that week (i.e., severe illness). 

#### 2.2.2. Food Addiction Symptoms

The Yale Food Addiction Scale (YFAS) [13] is a validated self-report instrument, and is currently the most frequently used measure of addiction-like eating behaviors. The original version of this scale was used in the current study. It includes 25 questions, with most presented in either Likert or “yes/no” format. Respondents are instructed to answer questions while keeping in mind “certain foods” for which they have difficulty controlling their intake. Each question contributes to one of seven symptoms (e.g., craving, failure to fulfill major role obligations) or clinical criteria (i.e., distress or impairment). The scale’s “diagnostic” threshold is based on the DSM-IV criteria for substance dependence (≥3 symptoms, plus distress or impairment). The questionnaire produces two metrics: a dichotomous “diagnosis” score (meets criteria vs. does not meet criteria) and a continuous “symptom” score (0–7 symptoms). The current study used the symptom score for analyses in order to better quantify the degree of change in symptoms.

The YFAS was administered at all five assessment points (Months 0, 4, 10, 16, and 22). While the original questionnaire asks participants to report on symptoms experienced in the past year, we modified the instructions to read “the past four months” at the Month 4 assessment and “the past six months” at Months 10, 16, and 22 in order to reflect the amount of time between assessments. 

#### 2.2.3. Body Weight

Body weight was measured to the nearest 0.1 kg using a calibrated digital scale at all five assessments. Participants wore light indoor clothing and emptied pockets prior to weighing. 

### 2.3. Recruitment and Initial Assessment

Recruitment to the parent study (Rural LEAP) primarily used mailed fliers and brochures presented at community and healthcare organizations. Screening occurred first via telephone and then in person at local county extension offices. At the in-person assessment, participants completed measurements of weight and height, psychosocial questionnaires (e.g., YFAS), and other health-/fitness-related assessments, such as a walking test [30]. Data collection occurred primarily through REDCap [36], which was approved by the University of Florida IRB for HIPAA compliance. 

### 2.4. Intervention

Participants were taught strategies to increase physical activity and reduce caloric intake. Each weekly in-person session consisted of a private weigh-in, reviewing progress, problem-solving challenges, a skills-based lesson, and setting of eating and activity goals. Lessons included topics such as basic nutrition education, physical activity, problem solving, managing cravings, seeking social support, and substituting high-calorie foods for low-calorie foods. All participants were instructed to continue self-monitoring during Phase 2. Details on the study and intervention content have been described in prior publications [30,31].

### 2.5. Statistical Analyses

Analyses were performed in SPSS version 26 and significance levels were set to *p* < 0.05. Multilevel modeling tests used the full sample (*n* = 182); other tests (e.g., descriptive analyses) did not include participants with missing data (see Figure 1 for sample sizes at each timepoint). One-way analyses of variance (ANOVAs), chi-squared tests, and Kruskal–Wallis tests were used for descriptive and supplementary analyses involving categorical variables. Pearson correlations and related-samples Wilcoxon signed-rank tests were used for analyses involving continuous variables. Bootstrapping to 5000 samples was used to correct for non-normal variables in parametric tests. Bootstrapping is a robust and effective resampling technique for non-parametric and/or smaller samples that does not make parametric assumptions on the distribution [37,38].

Multilevel modeling was used for Aims 1 and 2. The Aim 1 model included FA symptoms at each time point (Level 1) nested within 182 participants (Level 2). The model included the following Level 2 predictors: age, BMI, baseline problem food consumption (PFBaseline), Phase 1 changes in problem food consumption (PFChange), linear time slope (MonthLinear), quadratic time slope (MonthQuad), and the interactions between PFChange and each time slope. Level 1 predictors included: linear time slope (MonthLinear), quadratic time slope (MonthQuad), and BMI. Multilevel modeling also controls for the baseline value of the dependent variable (FA symptoms). In the equation below (Equation (1)), “FA” represents the dependent variable (FA symptoms) for person “*i*” at timepoint “*j*”. Terms marked with γ represent Level 2 intercepts as labeled after the underscore (e.g., γ_01__Age represents the Level 2 intercept for age). The residual is represented by the symbol “ζ_0i_”, and subsequent terms marked with ζ represent individual differences in Level 1 parameters not explained by Level 2 predictors. Age was included as a control variable in Aim 1 due to prior literature suggesting differences in food addiction symptoms by age group [8]. We originally added Phase 2 group randomization as a control variable in our multi-level models because it was significantly related to weight loss in the parent study (*n* = 445) [30]. However, it was ultimately removed from the current study due to worsened fit and no significant effects.

FA_ij_ = [γ_00__Intercept + γ_01__Age + γ_02__BMI + γ_03__ PFBaseline + γ_04__PFChange + γ_10__MonthLinear + γ_11__(MonthLinear*PFChange) + γ_20__MonthQuad + γ_21__(MonthQuad*PFChange)] + [ζ_0i_ + ζ_1i__MonthLinear + ζ_2i__MonthQuad + ζ_3i__BMI + ε_ij_] (1)

The Aim 2 model included body weight at each time point (Level 1) nested within 182 participants (Level 2). The model included the following Level 2 predictors: height, race, baseline problem food consumption (PFBaseline), Phase 1 changes in problem food consumption (PFChange), mean between-person FA symptoms (FAMean), centered within-person FA symptoms (FACent), linear time slope (MonthLinear), quadratic time slope (MonthQuad), and the interactions between PFChange, FAMean, and each time slope. The Level 1 predictors included: linear time slope (MonthLinear), quadratic time slope (MonthQuad), and centered within-person FA symptoms (FACent). Symbol definitions for Equation (2) are the same as for Equation (1). FA was split into Level 1 and Level 2 variables in order to test both within- and between-subject effects. Level 2 effects of FA symptoms were calculated by computing person-means (i.e., averaging each participant’s YFAS score across all timepoints). Level 1 effects were calculated by computing person-mean centered values (i.e., subtracting participants’ scores at each timepoint from their person-mean). Race was included as a control variable in Aim 2 due to prior literature showing differences in body weight by race [39].

Weight_ij_ = [γ_00__Intercept + γ_01__Height + γ_02__Race + γ_03__PFBaseline + γ_04__PFChange + γ_05__FAMean + γ_06__FACent + γ_10__MonthLinear + γ_11__(MonthLinear*PFChange) + γ_12__(MonthLinear*FAMean) + γ_20__MonthQuad + γ_21__(MonthQuad*PFChange) + γ_22__(MonthQuad*FAMean)] + [ζ_0i_ + ζ_1i__MonthLinear + ζ_2i__MonthQuad + ζ_3i__FACent + ε_ij_] (2)

One-way ANOVA and Bonferroni post-hoc tests were used to evaluate differences between mean values and change scores in FA symptoms and weight (respectively) at each timepoint. Median split graphs were also created to assist in interpreting significant quadratic time interactions. 

## 3. Results

### 3.1. Demographics

The final study sample included 182 participants (84.6% female) with a mean age of 55.4 ± 9.9 years. Demographic characteristics and YFAS scores are presented in Table 1. At baseline, 24 (13.2%) participants met the YFAS “diagnostic” criteria for FA, and the mean symptom score for the full sample was 2.38 ± 1.58. Non-white participants were more likely to have higher BMIs by about 1.5 kg/m^2^ (*p* = 0.031) compared to white participants. No other demographic variables were significantly related to YFAS scores or BMI. YFAS scores were not significantly related to BMI at baseline (*r* = 0.061; *p* = 0.410). There were no significant differences in demographic variables between Waves 1 and 2 (enrolled in the parent study before the YFAS was administered; *n* = 215) and Waves 3 and 4 (from which the current sample was drawn; *n* = 230, *p*s > 0.05). Additionally, there were no significant differences between our sample (*n* = 182) and participants in Waves 3 and 4 who were not eligible for the current study (*n* = 48; *p*s > 0.05).

### 3.2. Phase 1 Problem Food Consumption

Baseline problem food consumption was significantly associated with baseline FA symptoms (*r* = 0.230, *p* = 0.002). During Phase 1, the mean frequency of problem food consumption dropped from 2.5 ± 1.2 times/day to 1.5 ± 0.9 times/day (*p* < 0.001). Decreases in problem food consumption were significantly associated with Phase 1 reductions in FA symptoms (*r* = 0.165, *p* = 0.028), but not weight (*r* = 0.119, *p* = 0.115). However, correlation effect sizes were small. Phase 1 changes in FA symptoms were not significantly associated with weight change (*r* = 0.082, *p* = 0.277). 

### 3.3. Aim 1: Long-Term Changes in Food Addiction Symptoms

Five models (A–E) were tested using YFAS symptoms as the dependent variable and changes in Phase 1 problem food consumption as the independent variable. Body mass index, age, and baseline problem food consumption were included as covariates. Model A tested the unconditional means model, which included only the intercept. Model B tested the unconditional growth model, which added both linear and quadratic time slopes as predictors, both of which were significant (quadratic time, *p* = 0.012; linear time, *p* = 0.010). Model C tested for the fixed effects of baseline problem food consumption (covariate) and Phase 1 changes in problem food consumption (independent variable) on FA symptoms over time. Fixed effects test the association of a predictor with the intercept (in this study, baseline value) of the dependent variable. Neither predictor had a significant fixed effect.

**Figure 2:**
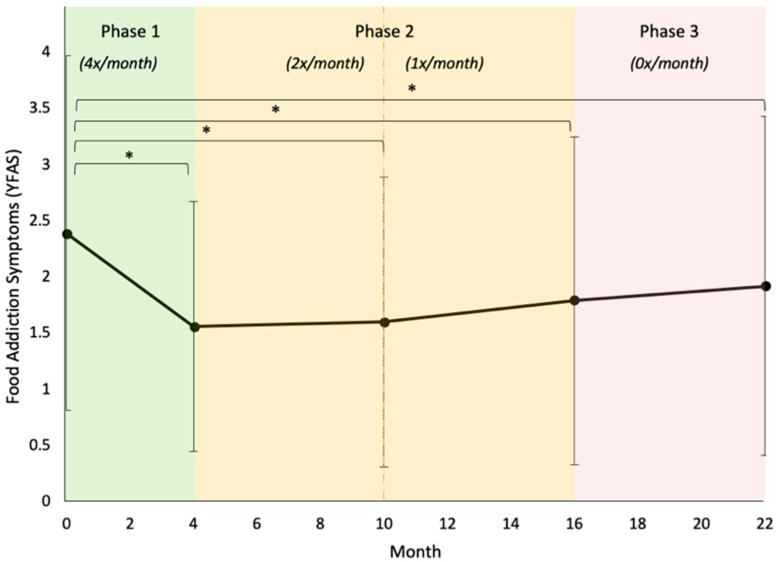
Model-implied trend of food addiction symptoms over time. Shaded areas mark Phases 1, 2, and 3 of the study, with the frequency of intervention sessions noted in italics. * = *p* < 0.05.

**Figure 3:**
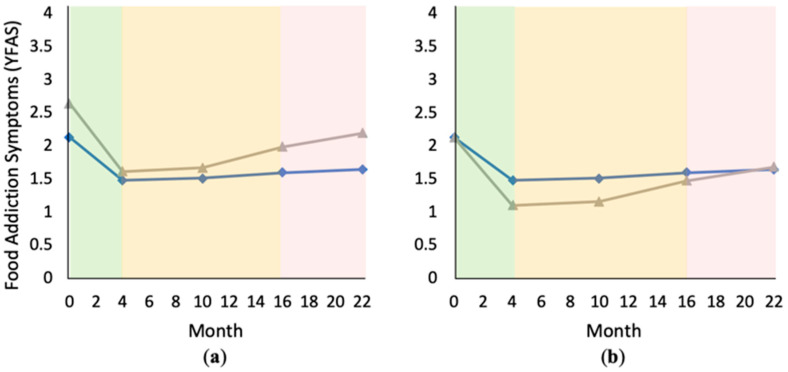
Changes in food addiction (FA) symptoms over time, comparing subjects with greater versus smaller Phase 1 reductions in problem food consumption (based on median split: greater reduction = gray triangles, smaller reduction = blue diamonds): (**a**) graph using original data; (**b**) graph correcting for baseline differences in Yale Food Addiction Scale (YFAS) scores for comparison.

Model D added the interaction of changes in problem food consumption with both linear and quadratic time, respectively. A variable’s interaction effect with time looks at its association with subsequent changes in the dependent variable across time (as opposed to at the intercept only). We found that the quadratic time interaction with problem food change was significant (*p* = 0.012), suggesting that Phase 1 decreases in problem food consumption were initially associated with a slightly sharper decline in FA symptoms, followed by a slightly sharper rebound. The interaction between problem food changes and linear time was not significant, indicating that Phase 1 problem food changes were not associated with long-term changes in FA scores. Model E included our remaining control variables (BMI and age), both of which had significant fixed effects (*p* < 0.001 and *p* = 0.043, respectively). The random effect of BMI was not significant (*p* = 0.321; see Figure 2 and Figure 3 and Table 2).

### 3.4. Aim 2: Long-Term Changes in Body Weight

Five models (A–E) were tested using body weight as the dependent variable and both FA symptoms and changes in Phase 1 problem food consumption as independent variables. Height, race (white vs. not white), and baseline problem food consumption were covariates. Model A was the unconditional means model and Model B was the unconditional growth model. In Model B, both linear and quadratic time slopes were significant (*p*s < 0.001).

**Figure 4:**
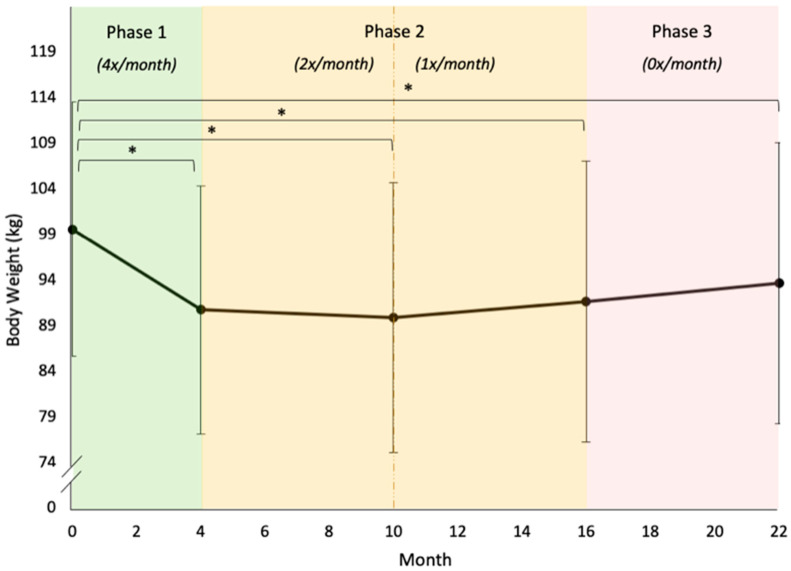
Model-implied trend of body weight over time. Shaded areas mark Phases 1, 2, and 3 of the study, with the frequency of intervention sessions noted in italics. * = *p* < 0.05.

**Figure 5:**
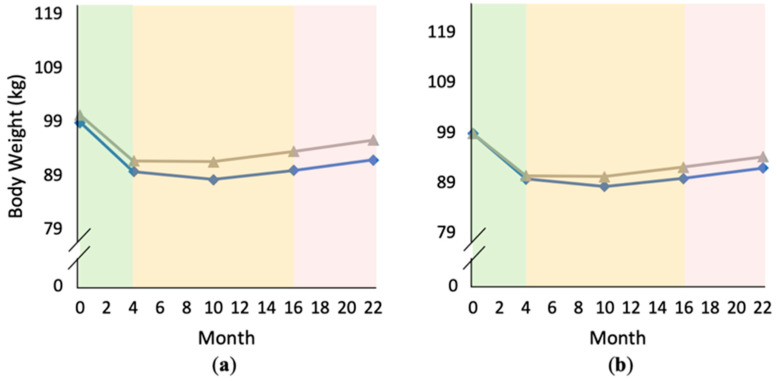
Changes in body weight over time, comparing subjects with higher versus lower person-mean YFAS scores (based on median split: higher YFAS scores = gray triangles; lower YFAS scores = blue diamonds): (**a**) graph using original data; (**b**) graph correcting for baseline differences in body weight for comparison.

Model C included the fixed effects of both FA symptoms and problem food consumption (baseline values and change scores); of these, only the fixed effects of within-person (Level 1, person-mean centered) FA symptoms were significant in Model C (*p* < 0.001). Model D added four interaction terms: Phase 1 changes in problem food consumption by time and person-mean (between-subject) FA symptoms by time. Of these, only the interaction between FA symptoms and linear time was significant (*p* = 0.008), such that higher mean FA scores were associated with less long-term weight loss. Model E added the remaining covariates (height and race), both of which had significant fixed effects (*p*s ≤ 0.001). The fixed effect of between-person (Level 2, person-mean) FA symptoms also became significant (*p* = 0.009) in this model, such that individuals with higher mean YFAS scores were more likely to have a higher baseline body weight. The quadratic time by person-mean FA interaction also became significant (*p* = 0.045), such that higher mean FA scores were associated with a slightly shallower weight-loss curve and a slightly steeper rate of weight regain (see Figure 4 and Figure 5 and Table 3).

### 3.5. ANOVAs and Bonferroni Post-Hocs: Food Addiction Symptoms and Body Weight

#### 3.5.1. Food Addiction

Finally, one-way ANOVA and Bonferroni tests detected statistically significant differences between baseline (Month 0) FA symptoms and each subsequent timepoint, respectively (all *p*s < 0.05). Months 4–22 were not significantly different from each other. Changes in FA symptoms between Months 0–4 (Phase 1) were significantly different from Months 4–10, 10–16, and 16–22 (first half of Phase 2, second half of Phase 2, and Phase 3, respectively; *p*s < 0.001). None of the subsequent phases were significantly different from each other (*p*s > 0.05).

#### 3.5.2. Body Weight

There were statistically significant differences between baseline (Month 0) body weight and each subsequent timepoint (all *p*s < 0.05). Months 4–22 were not significantly different from each other. Phase 1 changes in body weight were significantly different from all other timepoints (*p*s < 0.001), as were the changes during the first half of Phase 2 (Months 4–10; *p*s < 0.001; see Table 4).

## 4. Discussion

The primary goal of this study was to evaluate long-term changes in food addiction (FA) symptoms and weight loss among a sample of adults participating in a behavioral weight-loss program. We hypothesized that reductions in problem food consumption during the initial phase of treatment would be associated with long-term reductions in FA symptoms, that long-term changes in FA would be associated with long-term changes in body weight, and that initial reductions in problem food consumption would be associated with long-term reductions in body weight.

### 4.1. Food Addiction and Problem Foods

We found that changes in problem food consumption during the initial in-person treatment phase were not associated with changes in FA symptoms from the baseline to Month 22. Rather, reductions in problem food consumption were associated with a different pattern of change, suggesting sharper initial reductions in FA symptoms followed by a sharper rebound after the in-person phase. While future research is needed to clarify the relationship between problem food consumption and FA symptoms, our findings suggest that decreasing problem food consumption (in the context of a behavioral weight loss program) may be associated with improvements in FA symptoms in the short term, but that these changes may not last in the long term.

Many have proposed that, similarly to drug addiction treatment models, the goal of FA treatment should be either abstinence [40], moderation [23], or harm reduction [41] in regard to problem food consumption. Others have implied that non-dieting approaches may be beneficial [42,43,44,45]. However, few studies have tested the efficacy of any FA treatment. The FA studies identified by Cassin et al. [20] examined a variety of different approaches, including abstinence, cognitive-behavioral therapy, exposure and response prevention, intuitive eating, and mindfulness. However, only two of the included studies specifically targeted FA as a primary outcome. The authors concluded that more research is needed to compare the effects of potential interventions, particularly those drawn from evidence-based treatments for substance use or eating disorders [20].

### 4.2. Food Addiction and Weight 

We found that both FA symptoms and body weight decreased over time, and that higher average YFAS scores over the course of the study were associated with less weight loss. These findings are consistent with several studies that have shown an association between FA symptoms and body weight both cross-sectionally and across time [8,15,28]. However, others have failed to find an association [29,46,47]. Despite some inconsistency, most prior research has shown significant reductions in FA symptoms following weight-loss treatment [28,29,46,48,49]. These findings require further exploration, but it is possible that the connection between FA symptoms and weight may become more apparent over periods of time that extend beyond the length of typical intervention studies (i.e., 4–6 months), and/or that both FA and body weight are influenced or caused by an external factor.

### 4.3. Problem Foods and Weight

We did not find a significant association between early changes in problem food consumption and long-term changes in weight. This finding was contrary to our hypotheses, and also contrary to some prior cross-sectional and behavioral weight-loss studies [50,51,52,53,54]. One possible explanation could be that participants in our intervention were instructed to reduce calories rather than reduce problem foods specifically; the results may have been different had problem food consumption been directly targeted. Another possible explanation could be that our measure of problem food consumption (number of times consumed per day) did not provide enough power given its seemingly restricted range. However, at least one other study has reported a similarly unexpected finding: Nikiforova and associates [55] found that, among their sample of 300 laparoscopic sleeve gastrectomy patients, there was a 12.9% increase in the number of patients who reported a diet rich in “sweets” and an 8.9% increase in the number of patients who reported a diet rich in “snacks” three years after surgery, despite a significant reduction in BMI. They concluded that while consumption of these foods increased post-surgery, they did not appear to have a negative effect on BMI. Further research is required to clarify the association between high-fat and/or high-sugar foods and body weight, especially while controlling for caloric consumption.

### 4.4. Implications and Future Directions

Some have proposed that FA causes weight gain through the overconsumption of high-calorie foods (e.g., [56]), while others have suggested that obesity may cause FA symptoms via brain chemistry changes that lead to overeating (e.g., [57]). An additional question to consider is whether FA and obesity may both be symptoms of an external factor. For example, it has been well established that both obesity and FA symptoms are associated with increased depression and anxiety, lower self-esteem, and poorer quality of life [16,19,58,59,60,61,62]. In addition to psychosocial factors, biological contributors, such as gut microbiota and genetics, have also been associated with weight and addiction-like eating [63,64,65,66]. Future studies should prospectively analyze the relationships between neurobiological and psychosocial factors, body weight, and FA in order to predict which factors may put individuals at risk of developing either or both of these conditions. 

If FA leads to weight gain through the overconsumption of high-calorie, highly palatable foods, reducing consumption of these foods should lead to weight loss. However, the current study’s findings did not support an association between changes in problem food consumption and either concurrent (Phase 1) or subsequent (long-term) changes in body weight. Future studies should evaluate long-term changes in problem food consumption simultaneously with both FA symptoms and weight in order to elucidate these relationships. Future studies should also evaluate the long-term effects of problem food consumption on both body weight and FA symptoms by specifically targeting problem food consumption without caloric restriction.

### 4.5. Strengths and Limitations

The results should be interpreted in light of this study’s limitations. First, the analyses conducted cannot demonstrate causal relationships between the variables, nor determine the direction of the association between FA and body weight. Second, the study sample was primarily female and had sociodemographic characteristics similar to adults in the rural southeastern United States, which may limit generalizability to other populations. Additionally, the proportion of participants in the current study who met YFAS criteria for FA (13.2%) was less than the estimated prevalence for adults with overweight/obesity (approximately 25%) [8]. This may be due to the fact that ours was a treatment-seeking sample, as other behavioral weight-loss studies have also reported relatively low FA prevalence rates (e.g., 19% [15]; 6.7% [29]; 15.2% [47]). Third, dietary records were based on self-reporting and were not independently verified, and problem food consumption was coded post hoc for secondary data analyses. Additionally, we were unable to evaluate long-term changes in problem food consumption due to the low rates of self-monitoring after Phase 1. Fourth, the current sample size may have been insufficient to detect certain differences, such as the effects of Phase 2 group randomization and problem food consumption on weight change. Finally, the present results were consistent with previous research regarding the direction of change in problem food consumption [52,53,67,68]; however, differences in methodology (i.e., using measures of quantity vs. frequency) precluded our ability to compare the clinical significance of the change with that observed in prior studies e.g., [52,53,67,68]. Nevertheless, this is the first study to our knowledge that has evaluated long-term changes in FA symptoms in the context of a behavioral weight-loss program. The above findings regarding the associations between problem food consumption, FA symptoms, and body weight over time have important implications for future research.

## 5. Conclusions

Despite increased interest in the concept of food addiction, there is a dearth of research regarding its clinical implications and potential treatments. The current study examined long-term (two-year) changes in food addiction symptoms and their association with changes in body weight and “problem food” (i.e., highly palatable food) consumption among adults in a behavioral weight-loss program. The findings suggest that food addiction symptoms improve during behavioral weight-loss treatment and that reducing problem food consumption is associated with short-term improvements in FA symptoms. Despite some evidence for a negative association between food addiction symptoms and weight loss, more research is needed to elucidate the complex interplay of food addiction symptoms, body weight, and problem food consumption. A greater understanding of these relationships may inform the development of effective interventions for long-term changes in FA. 

## Figures and Tables

**Table 1:** Baseline sample characteristics (*N* = 182).

Characteristic	M (SD)	*n (%)*
Age (years)	55.4 (9.9)	
Weight (kg)	99.6 (13.9)	
BMI (kg/m^2^)	36.6 (3.6)	
YFAS symptom score	2.38 (1.58)	
YFAS clinical cutoff		
≥3 symptoms + distress/impairment		24 (13.2)
<3 symptoms or no distress/impairment		158 (86.8)
Gender		
Female		154 (84.6)
Male		28 (15.4)
Ethnicity		
Non-Hispanic		175 (96.2)
Hispanic		7 (3.8)
Race		
White		146 (80.2)
Black or African American		35 (19.2)
Hispanic or Latino		7 (3.8)
American Indian or Alaskan Native		6 (3.3)
Asian		1 (0.5)
Native Hawaiian or Other Pacific Islander		0 (0.0)
Highest level of education		
<High school		4 (2.2)
High school or GED		89 (48.9)
Associate’s degree		21 (11.5)
Bachelor’s degree		45 (24.7)
Advanced degree		23 (12.6)
Annual household income		
<$20,000		14 (7.7)
$20,000–$34,999		41 (22.5)
$35,000–$49,999		31 (17.0)
$50,000–$74,999		39 (21.4)
>$75,000		45 (24.7)
Unknown		12 (6.6)

**Table 2:** Aim 1 results of the model tests (food addiction symptoms as outcome).

	Par	Model A	Model B	Model C	Model D	Model E
**Fixed effects**						
**Initial status**						
**Intercept**	γ_00_	0.007 (0.058)	−0.216 ** (0.062)	−0.217 *** (0.061)	−0.135 * (0.062)	−0.134 * (0.062)
**PF Change**	γ_04_			−0.117 (0.080)	−0.026 (0.084)	−0.026 (0.085)
**PF Baseline**	γ_03_			0.088 (0.080)	0.098 (0.080)	0.088 (0.080)
**BMI**	γ_02_					0.262 *** (0.049)
**Baseline Age**	γ_01_					−0.113 * (0.056)
**Rate of change**						
**Time (linear)**	γ_10_		−0.082 ** (0.026)	−0.083 ** (0.026)	−0.040 (0.026)	−0.039 (0.026)
**X PF Change**	γ_11_				−0.003 (0.025)	−0.005 (0.025)
**Time (quad)**	γ_20_		0.214 *** (0.031)	0.215 *** (0.031)	0.135 *** (0.034)	0.135 *** (0.033)
**X PF Change**	γ_21_				−0.078 * (0.031)	−0.074 * (0.031)
**Random effects**						
**Level 1**						
**Residual**	ε_ij_	0.496 *** (0.027)	0.385 *** (0.028)	0.389 *** (0.028)	0.360 *** (0.026)	0.352 *** (0.028)
**BMI**	ζ_3i_					0.041 (0.042)
**Level 2**						
**Intercept**	ζ_0i_	0.503 *** (0.064)	0.468 *** (0.063)	0.437 *** (0.059)	0.443 *** (0.060)	0.420 *** (0.059)
**Time (linear)**	ζ_1i_		0.034 * (0.013)	0.034 * (0.013)	0.034 ** (0.013)	0.033 ** (0.013)
**Time (quad)**	ζ_2i_		0.042 * (0.017)	0.036 * (0.016)	0.041 * (0.016)	0.036 * (0.016)
**Fit Statistics**						
**Deviance**		2165.464	2099.093	2087.688	2046.568	2041.203
**AIC**		2171.464	2113.093	2105.688	2070.568	2069.203
**BIC**		2185.749	2146.424	2148.542	2127.693	2135.849

Estimates are presented with standard errors between parentheses. * *p* < 0.05, ** *p* < 0.01, *** *p* < 0.001. PF = problem foods; BMI = body mass index; AIC = Akaike information criterion; BIC = Bayesian information criterion.

**Table 3:** Aim 2 results of the model tests (body weight as outcome).

	Par	Model A	Model B	Model C	Model D	Model E
**Fixed effects**						
**Initial status**						
**Intercept**	γ_00_	0.003 (0.069)	−0.235 ** (0.070)	−0.228 ** (0.070)	−0.228 ** (0.070)	−0.230 *** (0.052)
**FA (M)**	γ_05_			0.083 (0.091)	0.105 (0.092)	0.181 ** (0.069)
**FA (C)**	γ_06_			0.101 *** (0.016)	0.101 *** (0.015)	0.101 *** (0.016)
**PF Change**	γ_04_			−0.107 (0.101)	−0.104 (0.101)	−0.063 (0.075)
**PF Baseline**	γ_03_			0.005 (0.100)	0.005 (0.100)	−0.015 (0.075)
**Height**	γ_01_					0.610 *** (0.052)
**White race**	γ_02_					−0.170 ** (0.052)
**Rate of change**						
**Time (linear)**	γ_10_		−0.123 *** (0.015)	−0.121 *** (0.015)	−0.120 *** (0.014)	−0.120 *** (0.014)
**X FA (M)**	γ_12_				0.051 ** (0.019)	0.050 ** (0.019)
**X PF Change**	γ_11_				0.002 (0.015)	0.002 (0.015)
**Time (quad)**	γ_20_		0.238 *** (0.013)	0.224 *** (0.013)	0.224 *** (0.013)	0.224 *** (0.013)
**X FA (M)**	γ_22_				−0.033 (0.017)	−0.033 * (0.016)
**X PF Change**	γ_21_				−0.005 (0.014)	−0.005 (0.013)
**Random effects**						
**Level 1**						
**Residual**	ε_ij_	0.147 *** (0.008)	0.049 *** (0.004)	0.044 *** (0.004)	0.044 *** (0.004)	0.046 *** (0.004)
**FA (C)**	ζ_3i_			0.002 (0.004)	0.001 (0.003)	0.001 (0.004)
**Level 2**						
**Intercept**	ζ_0i_	0.846 *** (0.092)	0.874 *** (0.093)	0.843 *** (0.090)	0.844 *** (0.090)	0.457 *** (0.050)
**Time (linear)**	ζ_1i_		0.030 *** (0.004)	0.028 *** (0.004)	0.027 *** (0.004)	0.026 *** (0.004)
**Time (quad)**	ζ_2i_		0.013 *** (0.003)	0.013 *** (0.003)	0.013 *** (0.003)	0.010 ** (0.003)
**Fit Statistics**						
**Deviance**		1443.129	1013.281	944.070	933.623	825.881
**AIC**		1449.129	1027.281	968.070	965.623	861.881
**BIC**		1463.536	1060.898	1025.195	1041.790	947.569

Estimates are presented with standard errors between parentheses. * *p* < 0.05, ** *p* < 0.01, *** *p* < 0.001. FA = food addiction symptoms; M = mean; C = centered; PF = Problem foods; AIC = Akaike information criterion; BIC = Bayesian information criterion.

**Table 4:** Means and change slopes for main variables by timepoint.

Timepoint M ± SD	YFAS Score	Body Weight (kg)	Timepoint M ± SD	Change in YFAS Score	Change in Weight (kg)
Month 0	2.39 ± 1.58 ^b,c,d,e^ *N = 182*	99.57 ± 13.95 ^b,c,d,e^ *N = 182*	Months 0–4 (Phase 1)	−0.882 ± 1.47 ^g,h,i^ *N = 178*	−8.83 ± 4.75 ^g,h,i^ *N = 182*
Month 4	1.56 ± 1.1 ^a^ *N = 178*	90.74 ± 13.56 ^a^ *N = 182*	Months 4–10 (Phase 2, 1st half)	0.044 ± 1.23 ^f^ *N = 161*	−0.789 ± 4.51 ^f,h,i^ *N = 180*
Month 10	1.60 ± 1.29 ^a^ *N = 164*	89.90 ± 14.8 ^a^ *N = 180*	Months 10–16 (Phase 2, 2nd half)	0.252 ± 1.10 ^f^ *N = 153*	1.93 ± 3.08 ^f,g^ *N = 174*
Month 16	1.79 ± 1.46 ^a^ *N = 163*	91.65 ± 15.38 ^a^ *N = 174*	Months 16–22 (Phase 3)	0.073 ± 1.11 ^f^ *N = 161*	1.80 ± 3.79 ^f,b^ *N = 174*
Month 22	1.92 ± 1.51 ^a^ *N = 177*	93.67 ± 15.38 ^a^ *N = 182*	-	-	-

Significance evaluated at *p* < 0.05 using One-way analysis of variance (ANOVA) and bootstrapped T-tests. Key: ^a^ = significantly different from Month 0, ^b^ = significantly different from Month 4, ^c^ = significantly different from Month 10, ^d^ = significantly different from Month 16, ^e^ = significantly different from Month 22; ^f^ = significantly different from Months 0–4, ^g^ = significantly different from Months 4–10, ^h^ = significantly different from Months 10–16, ^i^ = significantly different from Months 16–22.
